# Promoting equitable patient representation in therapeutic clinical trials by accounting for population disease burden

**DOI:** 10.1038/s41598-025-91174-x

**Published:** 2025-03-27

**Authors:** Megan Y. Gimmen, Kekoa Taparra, Curtiland Deville, Scarlett Lin Gomez

**Affiliations:** 1 Harvard Medical School, Boston, MA USA; 2 Department of Radiation Oncology, Stanford Health Care, Palo Alto, CA USA; 3 Department of Radiation Oncology and Molecular Radiation Sciences, Johns Hopkins University School of Medicine, Baltimore, MD USA; 4 Department of Epidemiology & Biostatistics, University of California, San Francisco, CA USA

**Keywords:** Medical research, Cancer, Biostatistics

**arising from**: I. Buffenstein et al.; *Scientific Reports* 10.1038/s41598-022-23664-1 (2023).

Buffenstein et al. reported an impressively comprehensive systematic review and meta-analysis of nearly 3000 randomized clinical trials of various diseases with over 600,000 participants between 2008 and 2019^1^. Using this data, clinical trial representation by gender, ethnicity, and race proportions were evaluated compared to the 2010 United States (US) Census^1^. We applaud the authors for including all five federally defined racial categories per the 1997 US Office of Management and Budget, including both the American Indian and Alaska Native (AI/AN) and the Native Hawaiian and Other Pacific Islander (NHPI) populations, who are often excluded or inappropriately aggregated in medical research^2,3^. The authors reported that NHPI and Black participants were overrepresented, while female, AI/AN, Asian, White, and multi-racial participants were underrepresented, with only female and Hispanic participant representation improving over time^1^.

When stratified by disease category and normalized to 2010 US Census data, the authors concluded that Black participants were underrepresented in only 4 disease categories but overrepresented in 20, while NHPI participants were not underrepresented in any category and overrepresented in 37^1^. In this study, the representation of each racial and ethnic group was measured by comparing trial proportions to the corresponding US population, without considering disease incidence^1^. Disease categories included broad groups defined by the researchers (*e.g.* infectious disease or neoplasms) and subcategories (*e.g.* hepatitis C or gynecologic cancer)^1^. However, disease incidence by gender, race, and ethnicity was not accounted for, which is known to vary considerably across diseases by population^4,5^.

By not adjusting for disease-specific incidence, representation calculations may yield misleading assessments of ethnic and racial representation in clinical trials^4,5^. This is because by overlooking disease-specific incidence, the actual need for disease-targeted interventions across different racial and ethnic groups remains unaccounted for. This potentially results in false perceptions of overrepresentation rather than reflecting disparities in disease burden and unmet clinical needs. In addition to concerns with the approach to normalization, trial data between 2008 and 2019 was compared to only the 2010 Census data^1^. Given the Census’s fixed 10-year interval, in contrast to the dynamic US racial and ethnic composition between 2010 and 2020, had the study accounted for these shifts in diversity, the analysis may have yielded different results.

Research investigating clinical trial representation has increasingly implemented an incidence-based approach to better contextualize trial inclusivity^5–7^. Varma, Gross, and Miller advocated that trial representation goals based on disease incidence can differ appreciably from those based on the national population^6^. For example, several studies reporting on oncology trial representation have used population-based cancer registry data as the source comparator^5,8^. Duma et al. calculated trial representation by an “enrollment factor” (EF), which they defined as the number of trial enrollees divided by the 2013 Surveillance, Epidemiology, and End Results cancer incidence^5^. These emerging studies serve as an example of how accounting for disease incidence is an important distinction and an alternative means of normalization for clinical trial representation analyses. Moreover, intentionally striving for robust clinical trial representation, particularly for minoritized populations, has the potential to build trust, promote fairness, and advance biomedical knowledge^7^.

While incidence-based clinical trial representation analyses may be challenging for some disease sites, data for select diseases exist to support its inclusion when available^5–7^. This data is critical to not only calculate accurate representation of minoritized populations in clinical trials, but to also contextualize the importance of meaningful overrepresentation to provide statistical power and analytical inclusion by clinical trialists when feasible^9^. Schwartz et al*.* find that even without aiming to identify subgroup-specific treatment differences, equitable representation in trials for diseases disproportionately affecting minoritized groups is essential due to the intrinsic importance of inclusivity and fairness in clinical research^7^. Moreover, for international trials, researchers may require non-US-incidence-based population benchmarks, emphasizing the need for more population-specific denominators. For example, researchers may seek representation goals beyond the geopolitical confines of the US, setting targets reflective of regional or global subgroup representation.

The authors reasonably framed the underrepresentation of Hispanic, AI/AN, Asian, and multiracial participants through the lens of historical injustice, socioeconomic status, and culture^1^. Paradoxically, the reported overrepresentation of Black and NHPI participants was explained by people of color being as willing to participate as White patients in clinical trials^1^. These findings were not challenged despite conflicting results in related studies showing the underrepresentation of Black and NHPI patients in clinical trials^8,10–13^. The authors state that disease-specific population demographics may explain overrepresentation for some racial and ethnic groups (*e.g.* higher Black participation in infectious disease, hypertension, stroke, and obesity trials)^1^. However, this same rationale was not extended to their findings of NHPI overrepresentation, despite reporting the NHPI population to be the most frequently “overrepresented” amongst included racial groups^1^.

As a medical research community, it is important to remember that there are lasting ramifications that persist downstream of reports of clinical trial overrepresentation and underrepresentation. For example, studies that conclude that Black and NHPI populations are overrepresented in clinical trials may unintentionally influence future clinical trial recruitment strategies, further exacerbating clinical trial representation against a path toward health equity, thus emphasizing the need to understand this relationship to inform culturally conscious incentivization and systemic intervention^6,7^. While enforcing federal racial reporting mandates is a key first step, achieving meaningful representation requires disease-specific incidence-based analysis and enrollment targets that ensure sufficiently powered data for minoritized populations disproportionately affected by certain diseases^6,7,9^. Expanding clinical trial access may also serve to promote trust within historically marginalized communities toward the drug development and approval process^7^. Thus, we do agree with and echo Buffenstein et al*.* for their advocacy for equitable representation within clinical trial participation. Ultimately, through advocating for incidence-based approaches to clinical trial enrollment goals and advocating for the collection of data that will help achieve these goals, researchers in the biomedical sciences can work towards developing clinical trials that are both representative and impactful for all communities.
